# Using Lessons Learned from Previous Ebola Outbreaks to Inform Current Risk Management

**DOI:** 10.3201/eid2105.142016

**Published:** 2015-05

**Authors:** Petra Dickmann, Andrew Kitua, Paul Kaczmarek, Julius Lutwama, Justin Masumu, Esron Karimuribo, Yunus Karsan, Nigel Lightfoot, Mark Rweyemamu

**Affiliations:** Dickmann Risk Communication DRC, London, UK (P. Dickmann);; Connecting Organizations for Regional Disease Surveillance, Lyon, France (P. Dickmann, P. Kaczmarek, N. Lightfoot);; Southern Africa Centre for Infectious Disease Surveillance, Morogoro, Tanzania (A. Kitua, E. Karimuribo, Y. Karsan, M. Rweyemamu);; Uganda Virus Research Institute, Entebbe, Uganda (J. Lutwama);; National Pedagogic University, Kinshasa, Democratic Republic of the Congo (J. Masumu)

**Keywords:** Ebola, Ebola virus, viruses, outbreaks, risk management, risk communication, infectious diseases, preparedness planning

Connecting Organizations for Regional Disease Surveillance (CORDS), together with the Southern African Centre for Infectious Disease Surveillance, organized an emergency meeting (September 1–2, 2014, in Dar es Salaam, Tanzania) to gather and collate first-hand experience from past Ebola outbreaks. The major aim was to identify key lessons that could inform current risk management. This meeting brought together a unique assembly consisting of scientists, policymakers, community and religious leaders, traditional healers, and media representatives from eastern and central Africa. They elucidated 3 major lessons that focus on improving communication, working with communities, and building and strengthening local capacity.

## Communication: Work with Communities, Not against Them

A major conclusion was that infectious disease management will work only when it is established with and within the community and not directed against it. This lesson requires community engagement in formulating infection control measures, as well as implementation, dissemination, and promotion of these measures. Infection control procedures are generally perceived as intrusive and, as such, often interfere with local social, cultural, and religious practices. For instance, the recommendation to avoid physical contact with a sick person is simply outside the behavioral norms in most communities. For example, from a community perspective, “protect yourself when caring for a sick person” would be a more suitable recommendation and a better alternative than a recommendation to avoid all physical contact. Building on this process of finding the right, appropriate containment measures, communication and health promotion work best when they involve community and religious leaders, traditional healers, and other advocates.

National and cross-border Ebola outbreaks are a new development, and engagement with various communities has presented a particular challenge throughout the current outbreak. A key aspect of this engagement is to devise and elaborate solutions for infection control that are consistent with local realities and practices. International health and aid organizations must strive to work in concert with communities to find adequate infection-control solutions.

## Communication: Share Early, Listen to Beliefs, and Read Rumors

Early sharing of information and surveillance data among professional groups was considered to lead to clear benefits. The countries of central and eastern Africa learned from previous outbreaks, and they have established infectious disease surveillance networks that operate in cross-border regions. Training in risk communication and One Health promote early communication among neighboring professionals across sectors and with the public. Multisectoral collaboration is key to early detection and faster response.

The risk communication approach also encourages public health professionals to engage with their communities to gain a better understanding of community values, opinions, and beliefs. For instance, a major lesson involved burial practices: participants explained that for local persons, a traditional burial is more essential than protecting themselves from an infectious disease they have not yet encountered. Therefore, the well-intended infection control messages went unheeded because they were not perceived to be relevant to completing a sacred ritual.

Traditional burial practices can hardly be stopped through the imposition of infection control measures (“Don’t touch/wash”), but they could be made safer by integrating protective steps into the rituals, such as using gloves and burying the deceased rapidly. International organizations might need to redesign their communication strategy to prioritize the communities’ needs, not their own requirements.

Communities have their own communication networks, and rumors spread fast and are influential. Regarding infectious disease, there are 2 kinds of rumors: 1) about possible cases (alerts) and 2) about community explanations of causes. Rumors distract from (compete with) the health message. Outcomes from this meeting suggest reframing rumors as useful guidance similar to pain, rumors provide often uncomfortable but useful feedback. The 2 main types of rumors are useful indicators to for 1) guiding case detection and 2) understanding where communication efforts go wrong. This reframing might enable health experts to detect cases earlier and to more effectively communicate with the public; it could also encourage a discourse about unusual disease events within communities.

Speaking from experience, public health experts advised communication specialists to consider using a case alert rumor book to provide alarm signals for quick response and follow-up response. In addition, a rumor book could serve as a record of the community explanations of infectious disease transmission and, as such, might help establish a starting point for community engagement. For instance, a common rumor was that Ebola is caused by witchcraft; the conventional response is to refer to Ebola as a virus. However, more useful would be to accept this alternative explanation and create recommendations consistent with community mind-set (e.g., don’t touch this person unprotected, but provide food [and prayers] as a token of empathy).

## Capacity Building: Avoid Blind Spots by Addressing the First Detectors

Cases typically appear before medical attention is sought and thus before cases can be understood as useful signals that trigger the activation of response. The initial detections of cases as alarm signals in a community represent the “blind spots” of capacity building: 1) capacity building on local level is often for health professionals, and 2) capacity building addresses a response mechanism (first responder training). Avoiding blind spots means shifting capacity building from reactive response to proactive detection involving the community.

Awareness raising and capacity building capacity at local levels must be continuous. For these purposes, a variety of training is now offered (World Health Organization and Centers for Disease Control and Prevention safety training). CORDS has designed an Intensified Preparedness Programme that offers short-term crisis response and longer term capacity building, consisting of training, advice, and background material for infectious disease management for affected and not-yet affected countries.

## Conclusions

The massive influx of international support addresses the foundational weaknesses of the public health systems. A key impairing factor, the engagement and communication with the community, is probably not yet addressed properly. The lessons learned in previous outbreaks identify major drivers of infection control as local realities and can advise the revision of the international response strategy with a focus on community engagement, communication, and capacity building. Effective community engagement during risk communication is a necessary and often underrated strategy to build trust and confidence for community health security and thereby global health security.

